# Prognostic value of vascular mimicry in patients with urothelial carcinoma of the bladder after radical cystectomy

**DOI:** 10.18632/oncotarget.12775

**Published:** 2016-10-20

**Authors:** Lin Zhou, Yuan Chang, Le Xu, Son Tung Nguyen Hoang, Zheng Liu, Qiang Fu, Zongming Lin, Jiejie Xu

**Affiliations:** ^1^ Department of Urology, Zhongshan Hospital, Fudan University, Shanghai, China; ^2^ Department of Urology, Ruijin Hospital, School of Medicine, Shanghai Jiaotong University, Shanghai, China; ^3^ Department of Biochemistry and Molecular Biology, School of Basic Medical Sciences, Fudan University, Shanghai, China

**Keywords:** vascular mimicry, bladder cancer, recurrence, radical cystectomy, adjuvant chemotherapy

## Abstract

Vascular mimicry (VM) refers to the plasticity of aggressive cancer cells forming de novo vascular networks, which promoted tumor metastasis. The aim of this study was evaluate the impact of VM on recurrence-free survival (RFS) in urothelial carcinoma of the bladder (UCB). Records from 202 patients treated with radical cystectomy (RC) for UCB at Zhongshan Hospital between 2002 and 2014 were reviewed. The presence of VM was identified by CD31-PAS double staining. Positive VM staining occurred in 19.3% (39 of 202) UCB cases, and it was associated with increased risks of recurrence (Log-Rank p<0.001). VM was identified as an independent prognostic factor (p=0.002). In the cohort with MIBC, patients with VM negative got CSS benefit from the use of ACT (p = 0.048). As for lung metastasis, the combination of VM and TNM stage (AUC 0.792) showed a better prognostic value than TNM stage alone (AUC 0.748, p = 0.008) or VM alone (AUC 0.714, p = 0.023). Vascular mimicry could be a potential prognosticator for recurrence-free survival in patients with UCB after RC. Vascular mimicry seems to predict risk of developing lung metastases after RC. The presence of VM identified a subgroup of patients with MIBC who appeared to benefit from adjuvant chemotherapy.

## INTRODUCTION

Urothelial carcinoma of the bladder (UCB) is the 7th most prevalent type of cancer in male population worldwide [1]. Radical cystectomy (RC) radical cystectomy is the standard therapy for muscle invasive and high risk non-muscle invasive bladder cancer patients [2]. Cisplatin-based combination chemotherapy is considered conventional first-line regimens for advanced UCB [3–5]. However, cisplatin-based chemotherapy has a low effective rate of 30%–40% [6]. Recent researches show that cancer stem cells get massive proliferation by enhanced survival after chemotherapy [7–9].

Tumor vascular mimicry (VM) refers to the formation of microvascular channels by tumor cells [10]. Several studies have shown that fluid can be drainaged by the PAS-positive tubular structures [11–13]. Further,Wang et al. [14] and Ricci-Vitiani et al. [15] provided powerful evidence that a part of the endothelial cells differentiated from cancer stem cells in the glioblastoma. In a study reported last year in Nature, Wagenblast et al. [16] discovered that Serpine2 and Slpi could stimulate the formation of VM and accelerate metastasis of breast cancer cells. A 2016 meta-analysis, which combined results from 36 studies on more than a dozen cancer types, estimated that patients' chances of dying were roughly doubled if their tumors showed evidence of VM [17].

In the present study, we evaluated the VM in UCB patients who underwent RC and explored its relation with recurrence-free survival (RFS), especially in patients receiving adjuvant chemotherapy after surgery.

## RESULTS

### Association of VM with clinicopathological features

**Figure 1:**
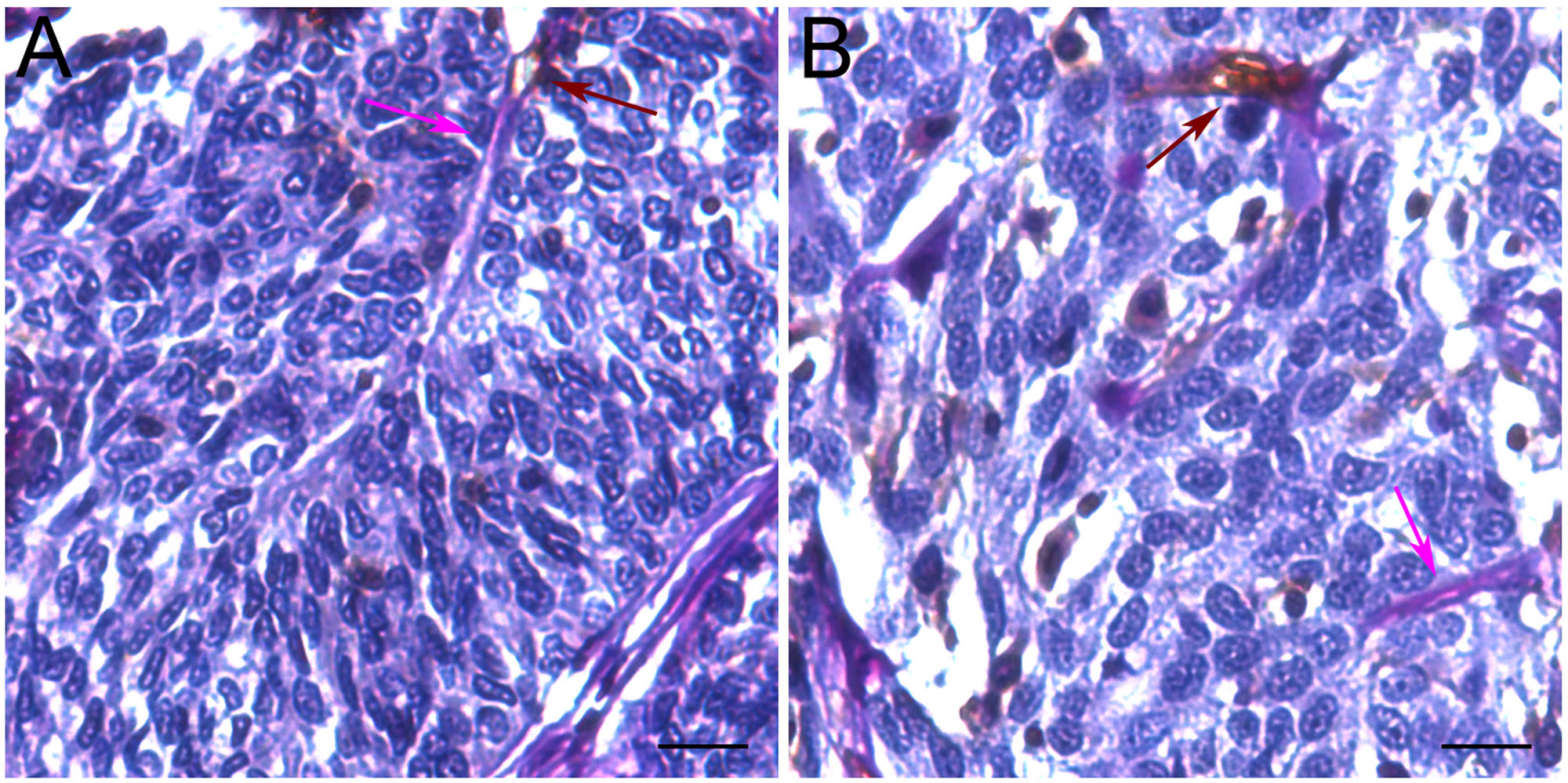
Vascular mimicry in UCB tissues Brown arrows indicate CD31+ endothelial blood vessels and pink arrows show PAS+/CD31- channels **A-B.** Scale bars (black lines) = 16.0 um.

The relationship between VM and clinicopathological features was shown in Table 1. The mean age of patients was 61.7 (SD±11.3) years and the mean Charlson comorbidity index (CCI) was 2.1 (SD±1.6). Eighty-nine (44.3%) patients had recurrence and 39 patients (19.3%) had positive staining of VM (Figure 1). Among these patients, 27 (13.4%) patients with recurrence had positive VM staining, and 62 (30.8%) patients with recurrence did not have positive staining of VM. In addition, positive staining of VM was significantly associated with pathological grade, pathological stage and recurrence (p = 0.035, p = 0.01 and p<0.001 respectively; Table 1).

**Table 1:** Association of VM with clinical characteristics in patients treated by radical cystectomy for urothelial-cell carcinoma of the bladder

	Patients	VM		
		Negative	Positive	p
**Gender**				0.243
**Female**	35(17.4%)	31(15.4%)	4(2.0%)	
**Male**	166(82.6%)	131(65.2%)	35(17.4%)	
**Age (years)**	61.65±11.28	61.17±11.56	63.64±9.91	0.263
**CCI**	2.13±1.61	2.10±1.65	2.28±1.41	0.307
**Pathological grade**				0.035
PUNLMP+low grade	48(23.9%)	44(21.9%)	4(2.0%)	
High grade	153(76.1%)	118(58.7%)	35(17.4%)	
**Pathological stage**				0.010
Ta, Tis, T1	59(29.4%)	54(26.9%)	5(2.5%)	
T2	91(45.3%)	74(36.8%)	17(8.5%)	
T3	30(14.9%)	21(10.4%)	9(4.5%)	
T4	21(10.4%)	13(6.5%)	8(4.0%)	
**Carcinoma in situ**				0.359
Absent	193(96.0%)	154(76.6%)	39(19.4%)	
Present	8(4.0%)	8(4.0%)	0(0.0%)	
**LVI**				0.074
Absent	98(48.8%)	84(41.8%)	14(7.0%)	
Present	103(51.2%)	78(38.8%)	25(12.4%)	
**Lymph-node metastasis**				0.105
Absent	191(95.0%)	156(77.6%)	35(17.4%)	
Present	10(5.0%)	6(3.0%)	4(2.0%)	
**Necrosis**				0.083
Absent	132(65.7%)	111(55.2%)	21(10.4%)	
Present	69(34.3%)	51(25.4%)	18(9.0%)	
**Surgical margin**				0.623
Negative	194(96.5%)	157(78.1%)	37(18.4%)	
Positive	7(3.5%)	5(2.5%)	2(1.0%)	
**Multifocality**				0.373
Unifocal	152(77.2%)	124(62.9%)	28(14.2%)	
Multifocal	45(22.8%)	34(17.3%)	11(5.6%)	
**Tumor size, cm**				0.287
<3	66(32.8%)	56(27.9%)	10(5.0%)	
≥3	135(67.2%)	106(52.7%)	29(14.4%)	
**Adjuvantchemotherapy**				0.262
No	119(59.2%)	99(49.3%)	20(10.0%)	
Yes	82(40.8%)	63(31.3%)	19(9.5%)	
**Recurrence**				<0.001
**Absent**	112(55.7%)	100(49.8%)	12(6.0%)	
**Present**	89(44.3%)	62(30.8%)	27(13.4%)	

Abbreviation: VM, Vascular Mimicry; CCI, Charlson comorbidity index; LVI, lymphovascular invasion; PUNLMP, papillary urothelial neoplasm of low malignant potential;

### Subgroup analysis of the prognostic value of VM in UCB patients after RC

**Figure 2:**
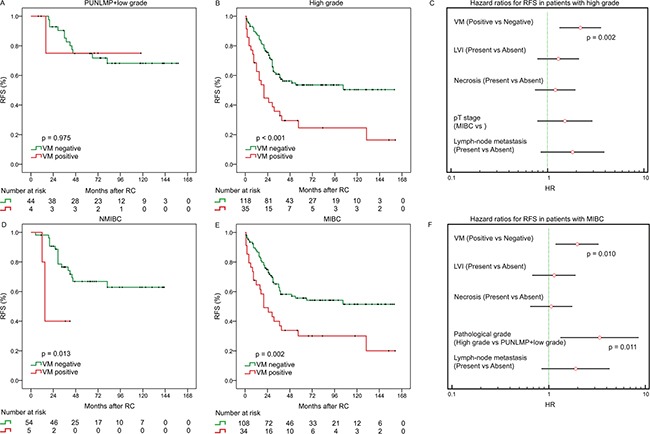
Subgroup analysis to assess prognostic value of VM by pathological grade and T stage Kaplan–Meier analysis of RFS in patients with PUNLMP+low grade **A.**, and with high grade **B.** Kaplan–Meier analysis of RFS in patients with NMIBC **D.**, and with MIBC **E.** Hazard ratios for RFS probabilities in patients with high grade **C.** and MIBC **F.**

Patients with positive VM staining had a lower rate of RFS compared with those with negative VM staining (p < 0.001; Supplementary Figure S1). The subgroup analysis by pathological grade showed that positive staining of VM was significantly associated with RFS in patients with high grade (p < 0.001; Figure 2B). Stratified multivariate analyses also showed an independent predictive impact of VM in patients with high grade for RFS (HR = 2.197, 95% CI 1.332-3.622, p = 0.002, Figure 2C).

In addition, the subgroup analysis by pT stage showed that positive staining of VM was significantly associated with RFS in NMIBC patients (p = 0.013; Figure 2D) and MIBC stage (p = 0.002; Figure 2E). Stratified multivariate analyses also showed an independent predictive impact of VM in patients with MIBC for RFS (HR = 1.971, 95% CI 1.178-3.299, p = 0.01, Figure 2F).

### Association between postoperative adjuvant chemotherapy (ACT) and VM

**Figure 3:**
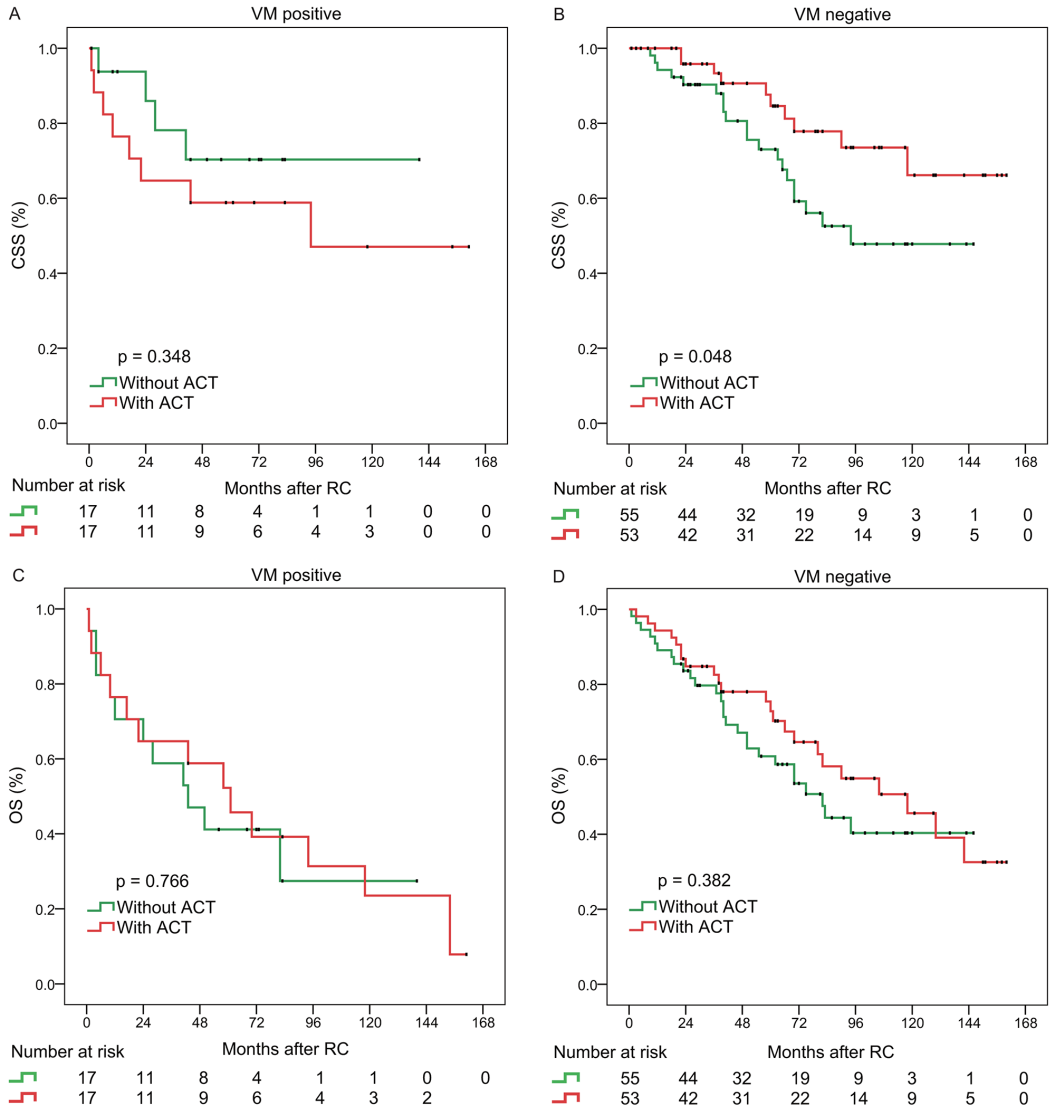
Relationship between VM and Benefit from Adjuvant Chemotherapy Kaplan–Meier analysis of CSS in patients with VM positive **A.**, and with VM negative **B.** Kaplan–Meier analysis of OS in patients VM positive **C.**, and with VM negative **D.**

Recent researches show that cancer stem cells get massive proliferation by enhanced survival after chemotherapy [7–9]. Wang et al. [14] and Ricci-Vitiani et al. [15] provided powerful evidence that a part of the endothelial cells differentiated from cancer stem cells in the glioblastoma. Therefore, we evaluated the benefit of platinum-based chemotherapy according to the presence of VM in patients who received postsurgical adjuvant chemotherapy. In the overall cohort, chemotherapy did not benefit patients regardless of VM status (Supplementary Figure S2). In the cohort with MIBC, patients with VM negative got CSS benefit from the use of ACT (p = 0.048) (Figure 3B).

### Prediction of pulmonary metastasis

**Figure 4:**
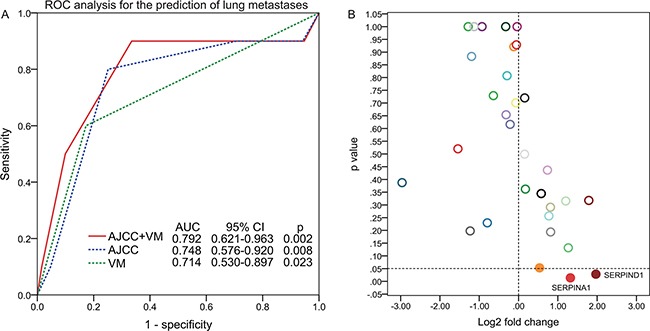
ROC analysis for the prediction of lung metastases **A.** The difference analysis for serpin family between UCB with lung metastasis and UCB without lung metastasis **B.**

The lung is one of the most common sites of distant metastasis from bladder cancer. [18] Some studies have shown that diagnosis of asymptomatic lung metastases of UCB increased survival rates [19, 20]. As for lung metastasis, the combination of VM and TNM stage (AUC 0.792) had a higher prognostic value compared with TNM stage alone (AUC 0.748, p = 0.008) or VM alone (AUC 0.714, p = 0.023) (Figure 4A). Recently, Wagenblast et al. [16] discovered that Serpine2 and Slpi could stimulate the formation of VM and accelerate metastasis of breast cancer cells. These two proteins were overexpressed preferentially in human patients that had lung-metastatic relapse. A recent study also implicated Serpins, including SERPIND1, in metastasis of breast cancer to the brain [21]. Based on TCGA data [22], we found that SERPIND1 (log2 fold change=1.97, p=0.028) and SERPINA1 (log2 fold change=1.32, p=0.014) were significantly upregulated in the group of UCB with lung metastasis compared with UCB without lung metastasis (Figure 4B).

### Construction and validation of prognostic nomogram for RFS

**Figure 5:**
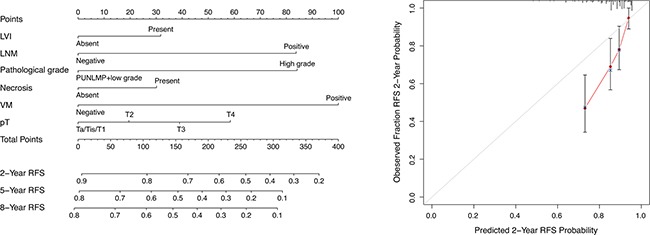
Nomogram for predicting RFS of UCB patients after RC Nomogram to predict RFS at 2, 5 and 8 years after RC (left). The calibration plots for predicting RFS at 2 years (right).

According to multivariate analysis of RFS, we built a nomogram model to predict RFS at 2, 5 and 8 years after RC (Table 2 and Figure 5). The calibration plots for our nomogram model are shown for predictions of 2-year RFS (Figure 5). In the overall cohort, the Harrell's C-index of the our nomogram was a slightly but insignificantly higher than that of BCRC nomogram [23] (0.678 vs. 0.642, p = 0.172). Similarly, the Harrell's C-index of our nomogram was slightly higher than that of BCRC nomogram among the patients with MIBC (0.666 vs. 0.617) and patients with high pathological grade (0.650 vs. 0.639) (Table 3).

**Table 2:** Univariate and multivariate Cox analysis of predictive factors of RFS

	Univariate analysis			Multivariate analysis		
	HR	95% CI	p	HR	95% CI	p
**Gender**			0.978			
**Female**	1	—				
**Male**	1.008	0.569-1.784				
**Age (continuous)**	1.012	0.994-1.030	0.211			
**CCI (continuous)**	1.052	0.938-1.181	0.387			
**Pathological grade**			0.002			0.055
**PUNLMP+low grade**	1	—		1	—	
**High grade**	2.550	1.413-4.601		1.840	0.986-3.434	
**Pathological stage**						
**Ta/Tis/T1**	1	—		1	—	
**T2**	1.532	0.897-2.615	0.118	1.058	0.595-1.881	0.847
**T3**	2.033	1.040-3.975	0.038	1.059	0.495-2.265	0.883
**T4**	2.850	1.416-5.735	0.003	1.627	0.756-3.499	0.213
**VM**			<0.001			0.002
**Absent**	1	—		1	—	
**Present**	2.516	1.599-3.959		2.156	1.325-3.510	
**Carcinoma in situ**			0.613			
**Absent**	1	—				
**Present**	0.743	0.235-2.351				
**LVI**			0.011			0.279
**Absent**	1	—		1	—	
**Present**	1.738	1.137-2.655		1.307	0.805-2.123	
**Necrosis**			0.021			0.351
**Absent**	1	—		1	—	
**Present**	1.651	1.080-2.524		1.251	0.781-2.003	
**Squamous differentiation**			0.777			
**Absent**	1	—				
**Present**	0.878	0.356-2.166				
**Surgical margin**			0.336			
**Negative**	1	—				
**Positive**	1.588	0.582-4.335				
**Lymph-node metastasis**			0.003			0.094
**Absent**	1	—		1	—	
**Present**	2.978	1.433-6.189		1.953	0.892-4.277	
**Multifocality**			0.774			
**Unifocal**	1	—				
**Multifocal**	1.076	0.653-1.774				
**Tumor size, cm**			0.913			
**<3**	1	—				
**≥3**	1.025	0.658-1.597				

Abbreviation: VM, vascular Mimicry; LVI, lymphovascular invasion; CCI, Charlson comorbidity index.

**Table 3:** Prognostication comparison of built-up nomogram and BCRC nomogram

	No. of patients	C-index			AIC	
		Nomogram	BCRC nomogram	p	Nomogram	BCRC nomogram
**Overall**	202	0.678	0.642	0.172	853.9	863.0
**MIBC**	142	0.666	0.617	0.223	612.9	621.7
**High pathological grade**	154	0.650	0.639	0.694	688.1	692.9

Abbreviation: C-index, concordance index; AIC, Akaike's information criterion; MIBC, muscle invasive bladder cancer.

## DISCUSSION

Although several anti-angiogenic agents have received U.S. approval for use in cancer patients, including Avastin and Nexavar, they only temporarily slow tumor growth; the tumors often become resistant. The evidence now suggests that vascular mimicry is one of the ways in which tumors develop a blood supply independently from classical angiogenesis [24]. The inner wall of VM consists of laminin, collagens, and heparin sulfate proteoglycans [10, 25]. Several studies provided strong evidence that fluid could be drainaged along the PAS-positive tubular structures that were surrounded by tumor cells [11–13]. Vascular mimicry may also promote metastasis, the migration of tumor cells to new parts of the body, which is responsible for most cancer deaths. The process of VM is controlled by complex signaling pathways including cancer stem cell, hypoxia, and angiogenesis related pathways [26]. These signaling pathways may be tractable as potential therapeutic targets and be good indicators for metastatic potential.

Our research mainly focused on the effect of VM on RFS in a large cohort of UCB patients (n=202) after RC. We found that positive rate of VM was 19.4% and in multivariate analysis VM resulted an independent risk predictor of RFS. Stratified Analysis showed that VM could help refine the risk stratification in the pathological grade or pathological T stage. Furthermore, our nomogram model slightly improved outcome prognosis than BCRC nomogram in patients with MIBC or high pathological grade. Therefore, our results indicated that VM could stimulate metastasis of UCB.

The most favorite sites of UCB metastasis are to lymph nodes, lungs, liver and bone [18]. Some studies have shown that diagnosis of asymptomatic lung metastases of UCB increased survival rates. [19, 20] The presence of VM may play an important regulating role in the metastasis of UCB, and may be used as a marker for predicting the potential metastasis. As for lung metastasis, the combination of VM and TNM stage (AUC 0.792) had a higher forecast precision compared with TNM stage alone or VM alone. As far as we know, currently there is no useful predictive tool for predicting lung metastasis of UCB.

More importantly, in the cohort with MIBC, patients with VM negative got CSS benefit from the use of ACT. This will be useful for better selection and management of patients who would receive ACT. According to the cancer stem cell (CSC) theory, at an early stage of systematic chemotherapy the majority of bladder non-CSCs are killed. However, a small proportion of bladder CSCs is selectively enriched by enhanced survival after chemotherapy, which results in local recurrence and distant metastasis [7–9].

The signaling pathways underlying VM offer new targets for tumor cell's stemness associated with metastasis and drug resistance. CVM-1118, a derivative of a plant compound, to curb the activity of Nodal, a gene that drives VM by making cancer cells more like stem cells [27]. In this year a phase I clinical trials was started to evaluate the safety of CVM-1118 in people with a variety of untreatable cancers and to assess its effectiveness [24].

Our study is limited by its retrospective design and the small sample sizes, which requires a multicenter, prospective study to validate. Given the heterogeneous nature of UCB and the population of our study, our conclusion might be overestimated and non-comprehensive due to these factors.

## CONCLUSION

Vascular mimicry could be a useful predictor of recurrence-free survival in UCB patients after RC. Vascular mimicry seems to predict risk of developing lung metastases after RC. The presence of VM identified a subgroup of MIBC patients who appeared to get benefit from adjuvant chemotherapy.

## PATIENTS AND METHODS

### Study population

This study was approved by the Clinical Research Ethics Committee of Zhongshan Hospital (Fudan University, Shanghai, China) and written informed consent was obtained from each patient. Records from 202 patients treated with RC for UCB at Zhongshan Hospital between 2002 and 2014 were reviewed and merged into a computerized database. Pathologic data included histologic type, tumor grade according to the 2004 World Health Organization classification [28], tumor and nodal stage according to the 2009 TNM classification [29], size of tumor, number of tumor, the presence of lymphovascular invasion (LVI), tumor necrosis, squamous differentiation and surgical margin status. A positive surgical margin was defined as the presence of tumor at the bladder and urethral and/or ureteral margin. LVI was determined according to the presence of carcinoma cells within an endothelium space. Patients whose chemotherapy regimen was recorded received platinum-based treatment. No patient had distant metastatic disease at the time of cystectomy. Recurrence-free survival (RFS) was defined as time from date of RC to local and/or metastatic recurrence, based on histologic or radiologic evidence. We calculated overall survival (OS) from the date of RC to the date of death from all causes. The cancer-specific survival (CSS) was defined as the interval from the date of RC to the date of death from UCB. During the entire study period, the follow-up protocol comprised history, physical examination, urine cytology, and laboratory measurements every 3–4 month in the first year, semi-annually in the second year, and annually thereafter. The follow-up period ended in June 2016.

### CD31-PAS double staining

Microarray development and immunohistochemistry were performed according to the methods previously applied [30]. Brifely, the slides were dewaxed in xylene and graded alcohols, hydrated, and washed in phosphate-buffered saline. After the endogenous peroxidase was inhibited by 3% H2O2 for 30 minutes, the slides were pretreated in a pressure cooker (3 minutes in sodium citrate buffer; pH = 6) and then incubated with 10% normal goat serum for 30 minutes. After overnight incubation at 4°C with primary antibodies (anti-CD 31 antibody, Code M0823, Dako, diluted 1/40), the slides were incubated with the secondary antibodies for 30 min. Reaction products were visualized by incubation with 3,3′-diaminobenzidine. The slides were then stained with Periodic Acid-Schiff (PAS) Kit (Sigma) according to manufacturer's instructions. PAS staining, haematoxylin and eosin staining, and CD31 immunohistochemistry were used to evaluate the presence and extent of mimicry as previously described [4, 13, 14]. Samples of UCB with high expression of VM formation served as the positive control. Negative control sections were treated with normal non-immune IgG instead of the primary antibody. Small vessel-like structures in the tumor that were PAS-positive but CD31-negative were presumed to be VM channels particularly if they contained red blood cells. All quantification was performed in a blinded setting.

### Statistical analysis

The chi-square test or Fisher exact was used for categorical variables, and the t-test or Wilcoxon rank-sum test for continuous variables. Kaplan-Meier analysis was used to determine RFS. Log-rank test was used to compare survival between subgroups. The Cox proportional hazards regression model was applied to perform univariate and multivariate analyses, and those parameters that demonstrated a statistically significant effect on RFS in the univariate analysis were included in the multivariate analysis. Harrell's index of concordance (C-index) and Akaike information criterion (AIC) were calculated to compare the accuracy of the prognostic model models, while Hanley-McNeil test was used to compare between C index [31, 32]. Statistical analyses were performed with SPSS, version 20.0 (IBM, Armonk, NY), Stata SE, version 13.0 (Stata, College Station, TX) and R software packages, version 3.1.2 (The R Foundation for Statistical Computing, http://www.r-project.org/). A two-sided *p* value of less than 0.05 was considered to be statistically significant for all reports.

## SUPPLEMENTARY FIGURES



## References

[1] Ferlay J, Steliarova-Foucher E, Lortet-Tieulent J, Rosso S, Coebergh JWW, Comber H, Forman D, Bray F (2013). Cancer incidence and mortality patterns in Europe: Estimates for 40 countries in 2012. Eur J Cancer.

[2] Witjes JA, Comperat E, Cowan NC, De Santis M, Gakis G, Lebret T, Ribal MJ, Van der Heijden AG, Sherif A (2014). EAU Guidelines on Muscle-invasive and Metastatic Bladder Cancer: Summary of the 2013 Guidelines. European Urology.

[3] von der Maase H, Sengelov L, Roberts JT, Ricci S, Dogliotti L, Oliver T, Moore MJ, Zimmermann A, Arning M (2005). Long-term-survival results of a randomized trial comparing gemcitabine plus cisplatin, with methotrexate, vinblastine, doxorubicin, plus cisplatin in patients with bladder cancer (Retracted article. See vol. 16, pg. 1481, 2011). J Clin Oncol.

[4] Saxman SB, Propert KJ, Einhorn LH, Crawford ED, Tannock I, Raghavan D, Loehrer PJ, Trump D (1997). Long-term follow-up of a phase III intergroup study of cisplatin alone or in combination with methotrexate, vinblastine, and doxorubicin in patients with metastatic urothelial carcinoma: A cooperative group study. J Clin Oncol.

[5] Sternberg CN, de Mulder P, Schornagel JH, Theodore C, Fossa SD, van Oosterom AT, Witjes JA, Spina M, van Groeningen C, Duclos B, Roberts JT, de Balincourt C, Collette L, Grp EG-UC (2006). Seven year update of an EORTC phase III trial of high-dose intensity M-VAC chemotherapy and G-CSF versus classic M-VAC in advanced urothelial tract tumours. Eur J Cancer.

[6] Shah JB, McConkey DJ, Dinney CPN (2011). New Strategies in Muscle-Invasive Bladder Cancer: On the Road to Personalized Medicine. Clin Cancer Res.

[7] Visvader JE, Lindeman GJ (2008). Cancer stem cells in solid tumours: accumulating evidence and unresolved questions. Nature Reviews Cancer.

[8] Clevers H (2011). The cancer stem cell: premises, promises and challenges. Nature Medicine.

[9] Kreso A, Dick JE (2014). Evolution of the Cancer Stem Cell Model. Cell Stem Cell.

[10] Maniotis AJ, Folberg R, Hess A, Seftor EA, Gardner LMG, Pe'er J, Trent JM, Meltzer PS, Hendrix MJC (1999). Vascular channel formation by human melanoma cells in vivo and in vitro: Vasculogenic mimicry. Am J Pathol.

[11] Clarijs R, Otte-Holler I, Ruiter DJ, de Waal RMW (2002). Presence of a fluid-conducting meshwork in xenografted cutaneous and primary human uveal melanoma. Invest Ophthalmol Vis Sci.

[12] Maniotis AJ, Chen X, Garcia C, DeChristopher PJ, Wu D, Pe'er J, Folberg R (2002). Control of melanoma morphogenesis, endothelial survival, and perfusion by extracellular matrix. Laboratory Investigation.

[13] Potgens AJG, vanAltena MC, Lubsen NH, Ruiter DJ, deWaal RMW (1996). Analysis of the tumor vasculature and metastatic behavior of xenografts of human melanoma cell lines transfected with vascular permeability factor. Am J Pathol.

[14] Wang R, Chadalavada K, Wilshire J, Kowalik U, Hovinga KE, Geber A, Fligelman B, Leversha M, Brennan C, Tabar V (2010). Glioblastoma stem-like cells give rise to tumour endothelium. Nature.

[15] Ricci-Vitiani L, Pallini R, Biffoni M, Todaro M, Invernici G, Cenci T, Maira G, Parati EA, Stassi G, Larocca LM, De Maria R (2010). Tumour vascularization via endothelial differentiation of glioblastoma stem-like cells. Nature.

[16] Wagenblast E, Soto M, Gutierrez-Angel S, Hartl CA, Gable AL, Maceli AR, Erard N, Williams AM, Kim SY, Dickopf S, Harrell JC, Smith AD, Perou CM, Wilkinson JE, Hannon GJ, Knott SRV (2015). A model of breast cancer heterogeneity reveals vascular mimicry as a driver of metastasis. Nature.

[17] Cao Z, Bao M, Miele L, Sarkar FH, Wang Z, Zhou Q (2013). Tumour vasculogenic mimicry is associated with poor prognosis of human cancer patients: A systemic review and meta-analysis. Eur J Cancer.

[18] http://uroweb.org/guideline/bladder-cancer-muscle-invasive-and-metastatic/−8.

[19] Giannarini G, Kessler TM, Thoeny HC, Nguyen DP, Meissner C, Studer UE (2010). Do Patients Benefit from Routine Follow-up to Detect Recurrences After Radical Cystectomy and Ileal Orthotopic Bladder Substitution?. European Urology.

[20] Volkmer BG, Kuefer R, Bartsch GC, Gust K, Hautmann RE (2009). Oncological Followup After Radical Cystectomy for Bladder Cancer-is There Any Benefit?. J Urol.

[21] Valiente M, Obenauf AC, Jin X, Chen Q, Zhang XHF, Lee DJ, Chaft JE, Kris MG, Huse JT, Brogi E, Massague J (2014). Serpins Promote Cancer Cell Survival and Vascular Co-Option in Brain Metastasis. Cell.

[22] Weinstein JN, Akbani R, Broom BM, Wang W, Verhaak RGW, McConkey D, Lerner S, Morgan M, Creighton CJ, Smith C, Kwiatkowski DJ, Cherniack AD, Kim J, Pedamallu CS, Noble MS, Al-Ahmadie HA (2014). Comprehensive molecular characterization of urothelial bladder carcinoma. Nature.

[23] Karakiewicz PI, Shariat SF, Palapattu GS, Gilad AE, Lotan Y, Rogers CG, Vazina A, Gupta A, Bastian PJ, Perrotte P, Sagalowsky AI, Schoenberg M, Lerner SP (2006). Nomogram for predicting disease recurrence after radical cystectomy for transitional cell carcinoma of the bladder. J Urol.

[24] Leslie M (2016). Tumors' do-it-yourself blood vessels. Science.

[25] Folberg R, Hendrix MJC, Maniotis AJ (2000). Vasculogenic mimicry and tumor angiogenesis. Am J Pathol.

[26] Kirschmann DA, Seftor EA, Hardy KM, Seftor REB, Hendrix MJC (2012). Molecular Pathways: Vasculogenic Mimicry in Tumor Cells: Diagnostic and Therapeutic Implications. Clin Cancer Res.

[27] Hendrix MJC, Seftor EA, Seftor REB, Chao JT, Chien DS, Chu YW (2016). Tumor cell vascular mimicry: Novel targeting opportunity in melanoma. Pharmacology & Therapeutics.

[28] Sauter GAF, Amin M, Eble JN, Sauter G, Epstein Jl (2004). Tumours of the urinary system: non-invasive urothelial neoplasias. WHO classification of classification of tumors of the urinary system and male genital organs.

[29] Sobin LHGM, Wittekind C (2009). TNM classification of malignant tumors.

[30] Xu L, Zhu Y, An H, Liu Y, Lin Z, Wang G, Xu J (2015). Clinical significance of tumor-derived IL-1beta and IL-18 in localized renal cell carcinoma: Associations with recurrence and survival. Urologic oncology.

[31] Harrell FE, Lee KL, Mark DB (1996). Multivariable prognostic models: Issues in developing models, evaluating assumptions and adequacy, and measuring and reducing errors. Statistics in Medicine.

[32] Hanley JA, McNeil BJ (1983). A method of comparing the areas under receiver operating characteristic curves derived from the same cases. Radiology.

