# Supplementary material for: Prognostic value of vascular mimicry in patients with urothelial carcinoma of the bladder after radical cystectomy
Source: Oncotarget. 2016 Oct 20;7(46):76214–23. doi: 10.18632/oncotarget.12775 (PMC5342808; doi:10.18632/oncotarget.12775)
Supplement: Supplementary file 1 [file oncotarget-07-76214-s001.pdf]

Prognostic value of vascular mimicry in patients with urothelial carcinoma of the bladder after radical cystectomy

SUPPLEMENTARY FIGURES

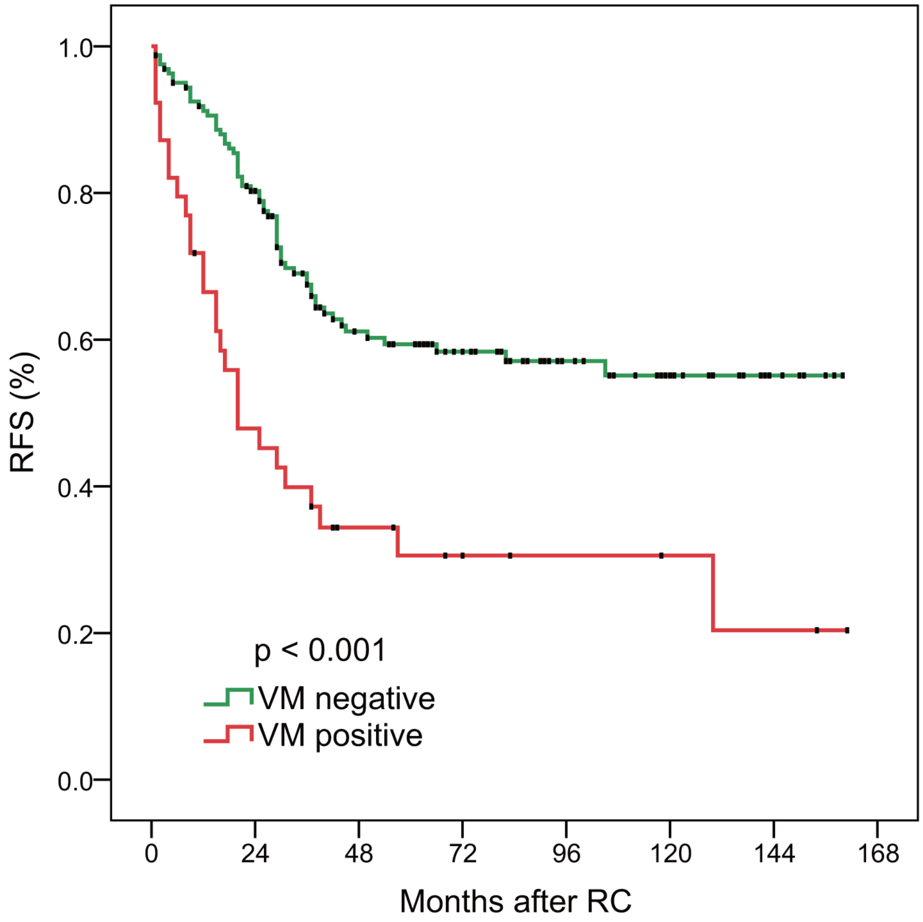

Supplementary Figure S1: Kaplan–Meier analysis of RFS according to the staining of VM.

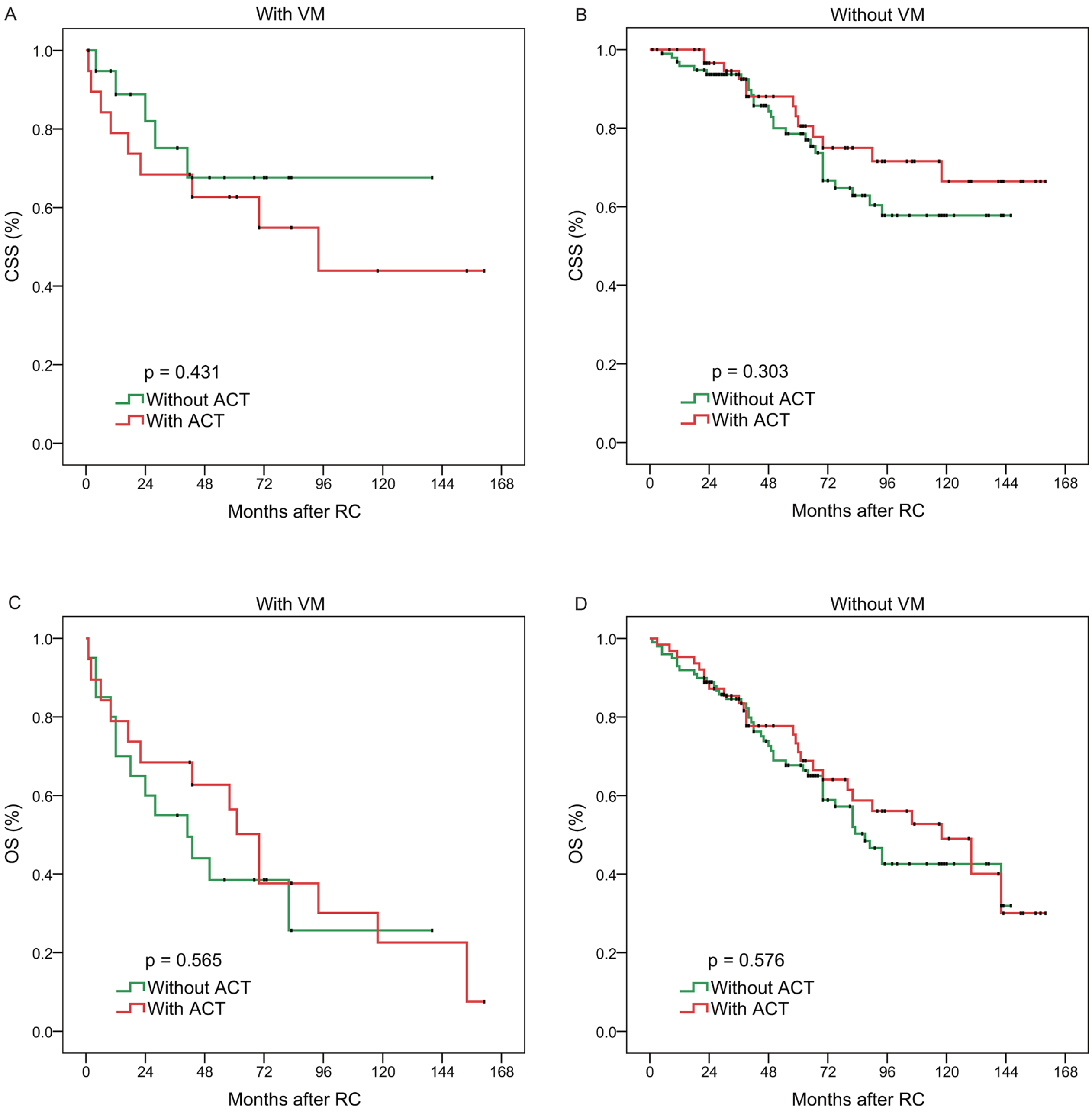

Supplementary Figure S2: Relationship between VM and benefit from adjuvant chemotherapy in the overall cohort. Kaplan–Meier analysis of CSS in patients with VM positive **A.** and with VM negative **B.** Kaplan–Meier analysis of OS in patients VM positive **C.** and with VM negative **D.**
